# Searching for Dwelling: Autism, Adolescence, and the Threat of “No Man’s Land”

**DOI:** 10.1007/s11013-025-09959-8

**Published:** 2026-01-19

**Authors:** Anne Toft Ramsbøl

**Affiliations:** VIVE – The Danish Center for Social Science Research and Aarhus University, Copenhagen, Denmark

**Keywords:** Critical phenomenology, Autism, Coming-of-age, Adolescence, Family life

## Abstract

What does it mean to come of age on the spectrum for autistic adolescents and their families? And how might this transitional stage be related to phenomenological questions of dwelling? As part of a broader research project on family life, autism, and coming-of-age in Denmark, this paper explores the case of the autistic adolescent Leo and his family to illuminate how families with autistic adolescents experience and respond to the coming-of-age process. Engaging with perspectives from anthropology of autism, disability, and critical phenomenology including the notion of dwelling, the paper demonstrates how coming-of-age poses pressing demands of (re)imagining, and searching for spaces of belonging and possibilities for becoming in both present and future horizons. These demands are often negotiated and shaped within a world that is dominated by neuronormative and chrononormative expectations, where the dynamics of misfitting are seldom a question of chance. I argue that families like Leo’s face not only practical and bureaucratic hurdles related to coming-of-age, but profound existential concerns. They face what I call an intensified dwelling problem of reimagining and searching for ways to feel at home in the world.

## Introduction

While typical kids will drive on the freeway, our kids will possibly be travelling other kinds of paths. Two of them will likely take the highway, a slightly slower path, yet somewhat parallel with the freeway. And our third kid, definitely the off-road, the bumpy, twisted path stretching through the wild forest with all kinds of detours. (Father of three autistic adolescents)

While coming-of-age for most adolescents is a liminal period of uncertainty and change, for autistic adolescents and their families, this period is often particularly fraught. Along with concerns for the future, many autistic^1^ adolescents face issues of co-morbidity, social isolation, and school absenteeism (PwC & VIVE, 2020), challenges that often intensify during adolescence (Bagatell, 2016). That navigating the coming-of-age process is a critical concern for families living with autism is reflected in the quote above, where a father of three autistic adolescents links their coming-of-age journey to a path that is neither straightforward nor smooth. Although this father represents a different family than the one discussed in this paper, his sentiment echoes the experiences of the family I will focus on and of several other families from my fieldwork on family life and autism—that autistic adolescents’ developmental paths are often filled with unexpected twists, off-road detours, and inevitable bumps along the way.

The quote above also highlights an alternative experience of coming-of-age—one that is framed in contrast to a dominant framework of typicality, where coming-of-age often is envisioned as a progressive, linear journey along a ‘freeway’ aligned with the fast-paced dynamics of modern society (Jensen, 2017). This normative trajectory of typicality is often defined by milestones such as gaining independence, pursuing an education, and entering the labor market (Ginsburg & Rapp, 2024; Grinker, 2020; Jensen, 2017). Families of children living with disabilities, however, are often compelled to create ‘new kinship imaginaries’ as atypical experiences reverberate through their life courses, raising fundamental questions of kinship, normalcy, and development—issues that become increasingly significant as the child transitions into adulthood (Rapp & Ginsburg, 2011). For many, this creates an existential gap between normative ideas of adulthood and developmental paths and the uncharted realities that living with disabilities often entail. Many families are especially concerned with where and how their child can receive the right care, feel a sense of belonging, and have opportunities to unfold and thrive (Ginsburg & Rapp, 2024). One might say that many families face an urgent demand to imagine, identify, and cultivate paths to and versions of feeling “at home in the world” (Jackson, 1995) that exceeds the normative and ‘typical’.

In this paper, I explore how families with autistic adolescents navigate the coming-of-age process along twisted, bumpy, and less-traveled paths and search for ways of feeling at home in the world. I do so primarily by exploring the case of Leo and his family, and subsequently by relating their experiences to those of other families who participated in a broader research project on family life, autism and coming-of-age in Denmark. I ask the following questions: How do families with autistic adolescents experience and respond to the pressing demands that the coming-of-age process entails? And how might the lived experiences of families with autistic adolescents teach us more about what it means to dwell or search for ways to dwell, when there is no simple way to dwell in present or future horizons?

As part of this exploration, I engage with the phenomenological notion of dwelling (Zigon, 2014). At first glance, dwelling and coming-of-age may appear to be separate topics. However, I aim to demonstrate that dwelling—understood as a particular way of inhabiting a world where one feels at home and where there are open possibilities for one´s being to unfold in that world (Zigon, 2014)—is a crucial and pressing concern for autistic adolescents and their families. Dwelling is thus not merely about the physical place one resides in or an ability to maintain one’s everydayness. Rather, dwelling encompasses how one creates and sustains a meaningful existence in a particular world (Zigon, 2014). For autistic individuals, this is particularly significant as they often navigate a world where neuronormativity is the “the dominant game in town” (Rodogno & Krause-Jensen, 2023, p. 237) that comprises chrononormative ideals and expectations of linear life course progression (Jensen, 2017) along a ‘freeway’ that is often not accessible to autistic individuals.

Zigon’s framework of dwelling stems from a still-emerging interdisciplinary scholarly field of critical phenomenology, which this paper positions itself within while simultaneously drawing on scholarship on autism and disability in anthropology and related fields. Like classical phenomenology, critical phenomenology is concerned with the conditions of possibility for experience and existence and takes its point of departure in the first-person perspective. Instead of focusing on transcendental (i.e., universal) features of experience as in classical phenomenology, critical phenomenology attends to the political, structural, and social embeddedness of experience—focusing on what (Guenther, 2021) has termed “quasi-transcendentals.” These are contingent, historically, and culturally sedimented structures which come to being in their quality of naturalness within specific life worlds—shaping the possibilities for lived experience and different forms of life. Calling upon this theoretical orientation, I examine the mechanisms through which such conditions shape the ways families living with autism experience and navigate adolescence and the coming-of-age process. Moreover, I draw on critical phenomenology’s engagement both with social critique—identifying “what is wrong” or problematic—and with how things might be “otherwise” (Guenther, 2021), or in other words, with the “otherwise” as a human potentiality (Zigon, 2014).

The paper is organized into three main analytical sections that explore the relationship between adolescence, coming-of-age, and the notion of dwelling from a critical phenomenological perspective. The first two sections examine two contrasting spaces: a school and a fire station. These environments provide radically different opportunities for Leo to inhabit, feel at home, and to unfold as he moves through adolescence. While both sections unfold in present time, I demonstrate how each space shapes Leo’s coming-of-age process and questions related to his future. The third section revolves around a bureaucratic meeting about the Leo’s transition to adulthood, and how his past and present experiences shape his coming-of-age process in practical, bureaucratic, and imaginative ways. This shaping is closely tied to his mother Mia’s struggle to keep him out of what she refers to as “No man’s land”—a metaphor she uses for a dreaded future horizon marked by non-becoming and non-belonging—a space of non-dwelling. I conclude by suggesting that Leo and his family’s case reflects broader issues faced by autistic adolescents and their families as they navigate the critical process of coming-of-age. This phase not only raises practical and bureaucratic questions but also imaginative, ethical, and existential ones—questions that the framework of dwelling helps illuminate. At its core lies the pressing demand of finding future ways of dwelling in inhabitable spaces that remain open to one’s being and unfolding.

## Case in Focus, Research Project, and Fieldwork

Empirically, this paper centers on one family—that of Leo, an 17-year-old boy—and the transitional issues that emerged during the time I followed them as part of my research on autism, family life, and coming-of-age in Denmark. This work included two years of ethnographic fieldwork with eight families with autistic young people. The young people in the families, aged between 15 and 22, broadly represent the diversity of the autism spectrum. While the ethnographic data unfolded in this paper illustrate the singularities of the life and transition of Leo’s family, many of their concerns, dilemmas, struggles, and hopes echo patterns across my fieldwork with families facing this transitional period. My close engagement with a single case is a deliberate commitment to the textures and subtleties of lived experience—to selves in formation, to emic expressions, and to the possibilities they offer for analytical scrutiny and theoretical reflection. I take these ethnographic insights as points of departure to explore broader societal and existential questions surrounding life with autism, family relations, and the coming-of-age process. In both Leo’s family and the other families, the fieldwork primarily took place within the family homes, with interviews and participant observation as the primary methods. Each visit was closely tailored to the preferences and possibilities of the family and young person. The fieldwork was approached with sensitivity to the fact that some autistic individuals may favor alternative modes of social engagement—such as forms other than direct face-to-face exchanges—and conversations with abstract or open-ended questioning (Ochs & Solomon, 2010; Rasmussen & Pagsberg, 2019). The family visits were supplemented by regular phone and e-mail conversations, participation in meetings between the families, schools, and municipalities as well as in network groups and courses for parents and grandparents of autistic children.

During my fieldwork, each family faced significant challenges in their children’s coming-of-age and transitioning to ‘adulthood.’ In Denmark, turning 18 triggers a significant bureaucratic transition for adolescents in the social service system, with changes in legislation and support options. Typical questions during this transition revolve around living arrangements, education, employment, finances, and legal status. These questions are often navigated in a context where many young autistic people face issues of co-morbidity and have encountered social and vocational difficulties in educational contexts throughout their school life—challenges which, for many, continue into adulthood, including within the available government-funded programs for autistic people (PwC & VIVE, 2020).

## Past and Present Dwelling Problems: School Misfitting

### Misfitting

I am like an *entirely* new type of LEGO box. I’ve always known there was something about me that wasn’t like the others (...) I started thinking poorly about myself. It got worse in situations where I was compared to the typical (..) when I wasn’t able to live up to normal standards. Eventually, I felt really bad, and I just burst like a balloon. (Leo)

One afternoon, I visit Leo and his mother, Mia. While we sit together on their living room couch, Leo shares his thoughts on being autistic, experiences with being different, and his ongoing struggles with school. Much of our conversation revolves around this school theme, and how Leo, for many years, has struggled to attend school and instead spends many days at home in his room. Leo’s difficulties with school began in the 5th grade: He started experiencing social challenges and was not thriving in what Mia describes as a “chaotic” school setting that could not respond to his needs. Leo’s experiences reflect those of several other young persons from my fieldwork. Research also highlights how school can be a significant challenge for autistic children and teenagers (Dumstrei, 2023; Fein, 2015b; Mattingly & Keeney Parks, 2022; Prince, 2010).

In another conversation I have with Mia, she shares that it took a “heavy fall”—years of increased emotional distress, frequent school absences, and ultimately a suicide attempt—before Leo was finally referred for diagnostic assessment, diagnosed with autism and ADHD at 16, and enrolled in a specialized school. While Leo appreciates his new school, describing it as a school that “can bend to your needs,” he still spends many days in his room, grappling with the lasting impact of his past school experiences—that is, the echoes of the burst balloon that he describes above.

As we see above, Leo has experienced a pervasive feeling of discomfort and being different within his school environment throughout most of his school life. These experiences echo the profound challenges many autistic persons face when navigating neurotypical-dominated environments (Fein, 2015b; Prince, 2010). Experiences of alienation—expressed through metaphors of living on foreign planets (Slomka in Rodogno & Krause-Jensen, 2023; Grandin in Sacks, 1996) or of not being “cut out for normal school and life” (Prince, 2010, p. 59)—reflect the profound disjunction that many autistic individuals, including the one’s I have followed as part of my fieldwork, experience between themselves and their environment.

In Leo’s account, this experienced difference is vividly expressed in his description of himself as an “entirely new type of LEGO box.” LEGO boxes, in their conventional form, are often associated with pre-defined shapes, structures, and expectations. They come with a set of bricks that are often assembled in ways that follow certain instructions. Leo, however, as an entirely new type of box contains bricks that do not easily align with sets of expectations and shapes of “the normal standards,” as he puts it.

This image and Leo’s other descriptions resonate with the notion of “misfitting” and “misfits” that have arisen in recent critical phenomenological work on disability (Garland-Thomsen, 2011, 2024). A misfit refers to the relational mismatch between an individual and a given environment or as the attempt to “fit some square pegs of content into some round holes” (2011, p. 5)—in other words, a relational disjuncture between two things that are forced together. This is a notion that emphasizes context and relation rather than essence and isolation—making disability a phenomenon that arises not solely from bodily (or in the context of the present paper, cognitive) difference but also from inaccessible or unaccommodating social and physical structures. Thinking about Leo’s metaphorical language of the LEGO box through this framework helps illuminate how Leo’s LEGO box metaphor reflects a misfit between Leo, the difference he embodies, and the standards and frameworks of typicality dominating the school environment.

Philosopher Erin (Manning, 2016) describes neurotypicality as a major key—a pervasive and structural tendency that organizes itself according to a predetermined set of values. Neurodivergence (e.g., autism), in contrast, is like a minor key—a variation on experience and a disturbing force to the major, “unmooring its structural integrity and problematizing its normative standards” (p. 1). While the rhythms and indeterminacy of the minor key hold great variational potential, they are however often cast aside, overlooked, or forgotten in the interplay of the major chords (p. 1). Manning’s image highlights not only how normativity operates, but also that, while everyone navigates shifting contexts of fit and misfit, experiences of misfitting are rarely a matter of chance. Some norms, structures, and their associated affordances are more deeply sedimented than others (Reynolds & Weiss, 2024). In the critical phenomenological framework, they hold a “quasi-transcendental” character by generating and consolidating meaning by “normalizing some habits of perception, cognition, and comportment while pathologizing others. In other words, they are not just phenomena in the world, but also (inter)subjective ways of seeing, hearing, moving, relating, and sensemaking” (Guenther, 2021, p. 6).

As we can see, misfitting is a persistent struggle for Leo. Over time, he begins to think poorly of himself, to feel “really bad”—illustrating how such sedimented ways of seeing and making sense shape not only how one is perceived by others, but also how one perceives oneself. For Leo, this is an experience that evolves, vividly illustrated by how he first describes himself as a LEGO box of pieces that lose their alternative, firm shapes, and then as turning into an inflated balloon that due to too much pressure dramatically bursts into multiple pieces. The image of the bursting balloon aligns with Mia’s description of a “heavy fall” and with the emic notion of “damaged goods” found in (Fein, 2015a, 2015b) work on autistic teenagers (p. 64). Damaged goods convey the idea of untapped potential resulting from disrupted schooling and social isolation, while highlighting the potentially harmful effects of not fitting and how an individual’s abilities and functioning are heavily influenced by the relation between person and context.

Leo’s metaphors of the LEGO box and the bursting balloon thus extend beyond differing from prevailing norms and standards or a relational disjuncture; they also highlight a fundamental challenge in unfolding, existing within and inhabiting a school world where there seems to have been no space to accommodate the unique forms and shapes that Leo represents. Leo’s metaphors also highlight a lack of open possibilities in which his atypical being can unfold. This is where (Zigon, 2014) critical phenomenological concept of dwelling can help illuminate the fundamental and existential dimension of Leo’s school experiences of misfitting, as I do below, and in a similar manner, issues related to coming-of-age on the spectrum, which I unfold in later sections.

### Entrapment: A Dwelling Problem

In everyday use, the notion of dwelling covers a house or place where one lives. In the phenomenological tradition of thought, the concept of dwelling (originating from Heidegger) addresses fundamental questions of what it means to ‘be-in-the-world.’^2^ In this perspective, dwelling is thus not merely about location habitation, it pertains to the existential experience of feeling ‘at home’ or truly ‘being’ in a particular environment where there are open possibilities for one’s being to dynamically unfold (Zigon, 2014).

Zigon (2014) develops his notion of dwelling through his work on anti-drug war political activism, exploring how anti-drug policies marginalize drug users, making them strangers in their own communities and their worlds increasingly uninhabitable. He argues that this ‘unsettledness’ highlights ethical^3^, existential, and ontological demands to be in the world in a certain way, noting that dwelling is not simply about being able to “live one’s everydayness,” as humans can live in all kinds of conditions (p. 756). Rather, to dwell is “to be in a world in such a way that one’s being is never pre-limited within a pre-assumed totality,”^4^ but where “possibilities for becoming otherwise remain open” (p. 757). In contrast, when someone is unable to dwell, their possibilities of ‘becoming otherwise’ are constrained. Dwelling, in this sense, speaks to both an ethical and existential inquiry into how people experience the world and how they can or cannot unfold within it.

Returning to Leo’s metaphor of the LEGO box, we see how it both depicts his struggles with misfitting while it simultaneously hints at a mode of potentiality. This entirely new type of LEGO box not only differs from typical kinds of LEGO boxes, it also holds the potential for an alternative form of ‘becoming’—metaphorically speaking, one that goes beyond the prescribed instructions of typical LEGO sets. Ethnographically speaking, it represents a potential process for Leo to develop as a person in ways that surpass dominant frameworks of neurotypicality. This speaks to the critical dimensions of the notion of ‘becoming otherwise,’ encompassing diverse human potentials—ways of being, thinking, and developing—how life and selves can exist and unfold beyond what is pre-conditioned by the rigid structures, norms, and expectations that govern a given society or social context.

What Leo’s descriptions of his school world illustrate, however, is an experienced lack of opportunity for him, as this LEGO box, to unpack its contents, to become ‘otherwise.’ Instead, Leo’s experiences of misfitting and of being measured against the framework of typicality and ‘normal’ standards speak to ways of being-in-the-world that align more with a kind of being that is “pre-limited within a pre-assumed totality” (Zigon, 2014, p. 757).

Another example of how Leo’s school experiences can be understood in terms of a dwelling problem is evident in the following passage where Leo, looking back at his time at school, especially emphasizes one recurring situation:

There were situations where I just stood there outside the door to the classroom for several minutes. I had my hand on the handle, and considered, “Should I go inside or not? Should I go inside or not?” This went on for a couple of minutes until I finally did it or gave up [and left]. (..) It was the feeling of shame! *Every time* I went into that classroom. The way they looked at me, stared at me.

Standing outside the classroom door with his hand on the doorknob, Leo hesitates, not sure whether he should enter or not. While his hesitation reflects an awareness that he will feel shame if entering, the awareness highlights the ‘pre’ aspect of both the pre-limiting and pre-assuming mechanisms that his school world represents for him. In this sense, the classroom door marks a threshold to a pre-defined world that he cannot inhabit or dwell in—it lacks the openness needed for his being to unfold. Instead, as we see, Leo faces a way of being-in-a-world that leaves him feeling wrong and shameful, confined within a dominant framework of typicality from which he can only deviate. In fact, whether Leo enters and faces the experience of estrangement, or leaves with the feeling of “giving up,” he ultimately faces a pre-limited kind of being. In this school world, Leo’s being is reduced to such a degree that it is more akin to “something like being trapped in a world” (Zigon, 2014, p. 757).

In another conversation, Mia describes how Leo, before even getting as far as the classroom door, would tear the car apart on their way to school, hit his head against the dashboard, or attempt to run back home once he got out of the car. Or, during the day, he would hide in a tree, a self-built den, or a playhouse. These descriptions further exemplify this sense of entrapment in a very literal way. Leo runs and hides from this world and ultimately retreats to his room for many days.

While Leo’s experiences with school may not explicitly address the theme of coming-of-age, they vividly reveal the baggage he carries with him as an adolescent approaching adulthood. They further demonstrate how his everyday life unfolds—how his developmental path largely takes place at home in his room, which I will later demonstrate inevitably shapes his possibilities and movements toward adulthood.

In this and the previous section, I have shown how a critical phenomenological framework can illuminate how, for autistic children and adolescents, the dynamics of misfitting can entail fundamental problems with being and unfolding, or dwelling, in neurotypical-dominated worlds—worlds that for many constitute the primary development context of childhood and adolescence outside the family home.

## The Fire Station: ‘Becoming Otherwise’ and Atypical Typicality

Even though Leo spends many days in his room in the period I spend with the family, there is one other place that he goes to regularly with great passion. Every week, he participates in a municipal firefighter program for teenagers who in one way or another face challenges. Leo describes this place with excitement and joy:

The others who come here also have ADHD and autism like me (..). With my firefighting friends, I don’t feel awkward. I try to be full of energy and kindness, say “good to see you again,” shake hands, and say “goodbye” when I leave. At least, I try to do that. It’s basic respect. It helps make it a positive and kind place. I also try to help people (..), pat them on the back. I’ve become the person that people ask for help. I just want to make it fun for everyone, help, and show that I take things seriously.

In contrast to Leo’s school world, the fire station provides a space where he fits—and not misfits—in a fundamental and different way. Mia describes this fit in terms of the material environment where everything is in order. As she says, “there’s no mess, they have racks and cups with their names on (..). Everything is planned, A, B, C. Everything has its place.” As we see above, Leo emphasizes the importance of the social environment—a space where, unlike the school world that often overwhelms him with shame and hesitation, he enters with “energy and kindness.” In contrast to standing hesitantly with his hand on the classroom doorknob, at the fire station he actively marks his presence and social belonging by greeting others with a “hello” and “goodbye” as he enters and leaves.

During our conversation, Leo laughingly describes how he and one of his “autistic friends” at the program bond over their shared habit of sitting in a specific position when they are playing computer games (he demonstrates how they like to sit slightly tilted with their legs up—echoing the potential of autistic social spaces, where forms of connection, senses of belonging, and positive identities can be cultivated if otherwise dominant neurotypical standards and expectations are not dominating (Bagatell, 2010; Fein, 2015a, 2015b)). Such communities are often structured around shared interests and alternative forms of sociality where traits that are often associated with autism are shared and valued instead of treated as problematic symptoms. The potential of such communities must be understood in light of the persistent struggles many autistic persons face when navigating neurotypical-dominated environments.

In the firefighting program, Leo neither feels confined by neurotypical norms nor does he experience the discomfort of being “awkward” and of not fitting. Instead, this is a place where atypicality is the typical. Notably, Leo also emphasizes the opportunity to cultivate virtues that he cherishes, such as kindness, responsibility, and engagement. He can demonstrate his ability to take things “seriously,” as he puts it, and has become a trusted figure within the group—someone who helps others and actively contributes to a positive social environment.

Considered in terms of dwelling, Leo’s account demonstrates that *becoming otherwise* is about being able to cultivate a particular kind of selfhood and sustaining a dynamic way of being. His descriptions also reveal that this unfolding is inseparable from a sense of fitting in and belonging within a particular social community—one that carries open possibilities for him to be and to unfold. For Leo, dwelling thus also entails being part of a social space where he thrives, giving him the sense of being at home with others within specific material surroundings.

In his work, Zigon elaborates on the notion of ‘becoming otherwise’ as a way of being-in-the-world where one remains intimately intertwined with and concerned for one’s environment and its constituent parts. This is an ongoing process of “building and maintaining a world in which such dwelling always remains possible” (Zigon, 2014, p. 757). Leo’s description of his experiences at the fire station reflects a process of his ‘becoming otherwise’ as he shapes and reshapes the social space into one where he and others can dwell, a process that contrasts with Leo’s non-dwelling in the school world, characterized by absence, hiding, and hesitancy.

### Future Horizons of Becoming: “Then What Can I Become?”

During my fieldwork with Leo’s family, Leo starts and completes the firefighter program through immense effort and perseverance. While the fire station offers Leo a space to dwell in the present, it also opens up a horizon for a possible future closely tied to his concerns about coming-of-age, aligning with his newly discovered experience of being able to demonstrate his ability to take things “seriously.” As the notion of ‘becoming otherwise’ suggests, dwelling is not merely about an ability to inhabit a given space in the present. It also implies an orientation to the future, of having future possibilities to be and to unfold. In this and the following sections, I will focus on the question of the future and on, as alluded to in this paper’s opening quote by a father of three autistic children, what it means to travel along bumpy and twisted off-road tracks. For Leo, as well as for many other autistic adolescents and their families, the future and coming-of-age is fraught with uncertainty (Ginsburg & Rapp, 2024).

I have found this goal [becoming a firefighter] after spending hours, months, days, and weeks worrying about what I can become when I’m older. I can’t imagine sitting at a cash register at the supermarket, nor can I sit at a desk in an office, I just wouldn’t be able to. So, I’ve spent so much time thinking, going crazy: “then *what* can I become?” (Leo)

Leo is aware that he will not thrive in what he considers to be a typical job. Instead, he has been haunted by the question, “then *what* can I become?.” This profound uncertainty of not meeting society’s common-sense imaginaries of coming-of-age echoes the metaphor that opens this paper: for some, the ‘freeway’ of normative development is simply not an accessible path.

As Leo says, “I can’t compare myself to ‘normal life’ and therefore, I don’t know what to expect as I grow older.” Leo’s contrast between ‘normal life’—the life that seems to follow an imaginable path toward securing a job at a supermarket or working behind a desk in an office—and the absence of any clear imagination of what his own future could entail, reveals how coming-of-age and the transition to adulthood are often dominated by frameworks of typicality and normative assumptions about education, employment, and independence (Ginsburg & Rapp, 2024)—frameworks that work in quasi-transcendental ways (Guenther, 2021).

Using dwelling as a concept that also holds a future dimension is particularly useful when considering Leo’s situation, where urgent demands about the future emerged for him and his family during my fieldwork. Leo’s dwelling issues extend beyond school and firefighting; they are deeply tied to his future imaginaries and to searching for ways to dwell in the future with open possibilities for him to unfold. In this sense, his question “then *what* can I become?” speaks not only to a query about possible employment but also to a deeper existential concern, one that speaks to the very possibility of dwelling in the future.

## The Bureaucratic Scene: Navigating the Pressing Demands of Coming-of-Age

About a year after my conversation with Leo and Mia on the couch in their living room, I accompany Mia to Leo’s coming-of-age meeting at the local municipality. As I witnessed was the case for several other young people’s coming-of-age meetings, the young person in question—Leo in this case—is not present. “This whole coming-of-age process is too overwhelming for Leo,” Mia explains, “I don’t want him to know how much is going on in the machinery behind him.”

As noted earlier, in Denmark, adolescents in the social service system transition from youth to adulthood when they turn 18 in bureaucratic terms. This involves moving from the jurisdiction of one set of legislation to another, with ensuing changes in available services and programs, which requires families to navigate urgent questions about education, finances, housing, and legal status. For Leo, turning 18 means that he will age out of both his school and the firefighting program. Prior to the meeting, Mia shares her concerns:

I’m on shaky ground (..) The educational advisor is practically a judge of what we are offered. It seems like the consequence of bad school options over time is now coming into force (..). I’m shocked that we are looking into a scenario where he might not be referred to *anything* at all.

In recent years, Mia has “come to terms,” as she says, with a future horizon that no longer aligns with traditional developmental pathways. Recently however, she has discovered that Leo may not be eligible for the public specialized educational program for young adults who need alternative pathways. To join this program, one must be able to “prove,” as Mia explains, regular school attendance, which, as demonstrated, has been a great challenge for Leo. The possible scenario of Leo transitioning from his present school to no school or “transitioning to nowhere” (Ginsburg & Rapp, 2024, p. 120) comes as a shock to Mia. In response to this, she states with great conviction that her goal for the meeting is to leave with a “plan.” This is a concrete example of how families living with disabilities are often faced with urgent demands of reimagining the course of life, particularly as their children come of age (Ginsburg & Rapp, 2024). Mia’s concern also demonstrates how such reimaginations are partly conditioned by the working “machinery” and limits of the bureaucratic system. In the following, I provide ethnographic excerpts from the scene that unfolded during the meeting:

In the meeting room, Mia reports on the family’s current situation. She tells the representatives from the municipality that Leo is still finding it hard to leave his room in the morning to go to school.

“It’s hard to think about him coming of age. He knows, he isn’t ready (..). I have this knot in my stomach (..) so far, it hasn’t gone very well (..) With this transition, there’s a great risk that he will fall heavily again,” Mia says, referring to the “heavy fall” mentioned earlier: a culmination of emotional distress, school absenteeism, and Leo’s suicide attempt.

“Leo has got what it takes when he is met the right way,” she then adds. She mentions the firefighting program. Even though Leo has aged out of the program, he has become part of the volunteer firefighting force and is sometimes called in to assist. “*This*, he is doing really well. He happily stays half an hour extra,” Mia says.

One of the municipal participants, Ellen, says that Leo categorically fits the special education program. However, his irregular school attendance raises two concerns: whether he can handle the program and whether the municipal committee will approve him to the program. The conversation goes back and forth.

“But there needs to be a *context* in which he can get ready!” Mia eventually says. “He won’t get ready at home. He already feels he is being excluded, lost (..). He feels ashamed when his classmates ask him what he’s going to do after the summer break. He is very much aware that he’s facing No man’s land.” Her cheeks are blushing with frustration.

Participants from various municipal departments explore what other options Leo has: “Could Leo get some kind of job at the firefighting program?,” “Can he continue at his school?,” “(..) are there any other possibilities for where he can go?”

“No”, “It would have to be approved by the visitation board, I don’t think so”, “No”.

Mia bursts out: “I'm confused at a higher level. There must be some sort of shelf if you can’t get into the program? Isn’t there something *else*?”

One of the participants explains that Leo can begin the program later, adding that if he “ends up” receiving disability pension, he would have access to various activities.

The conversation then turns to Leo’s current and future living situation. Mia is asked what she thinks. She stares into space. She then looks up and says resolutely:

“I don't think we finished discussing the other thing. We’re *still* in No man’s land.”

Suddenly, the scheduled time has run out. Laptops are being packed into backpacks and papers stacked together. Mia, however, stays put and runs through her stack of papers. While getting up from her chair, Ellen, one of municipal representatives says:

“If we agree that I recommend [Leo for] the program to the municipal board, I’ll do that?” She looks at Mia, awaiting her response. Mia looks relieved.

Mia: “I’d like that. Then, we have something to look forward to, we’re in safe hands.” Ellen hesitates. She nods, grabs her things, and leaves.

### Coming-of-Age as a Dwelling Problem and the Threatening Horizon of ‘No Man’s Land’

I now explore the emic notion of ‘No man’s land’ that emerges in the above ethnographic description of the bureaucratic scene. I examine how the notion reflects an urgent demand to respond to an intensified dwelling problem of the future. During my fieldwork, the metaphor of ‘No man’s land’ emerged in several families. Originally referring to a desolate area that no one controls, inhabits or owns, often in the context of war, the metaphor often covers situations of being lost and stuck in unruly contexts. At the bureaucratic coming-of-age meeting, Mia employs the metaphor to illustrate the potentially fraught future Leo is facing. Unlike his classmates and peers who are pursuing various educational paths toward envisioned futures, Leo faces the future of a ‘No man’s land’—the place of no future educational plans, or imaginable paths to develop.

In another family, a father describes ‘No man’s land’ in similar terms:

“Well, there are these standard packages, and if you fit into them, they have something to offer you. But if you don’t, it’s like you fall out of the frame and end up somewhere in No man’s land.”

This notion resonates with a broader concern shared by several families: securing a future for their child where they are not only met with care, but where they can thrive and unfold. “She shouldn’t go somewhere *just* to be taken care of,” a mother from another family states about her daughter. This care aspect of coming-of-age is also crucial for these families. In addition to using the metaphor of ‘No man’s land’ in the context of education, Mia and her husband also apply it to describe the feared situation in which parents of children with special needs are unable to care for their children, thus leaving this responsibility to the state. In this context, Mia expresses the fear that there may be no future place outside their family home capable of “containing” Leo, as she puts it. In this sense, ‘No man’s land’ speaks not only to haunting questions of possible ‘becoming’ but also to issues of both care and belonging. How can one possibly find care and a sense of social belonging in a No man’s land?

“Getting a plan” is not only a practical matter for Mia. It also refers to an existential concern about finding ways to dwell in the future when there is no way simple way of doing so. Mia’s words “I’m on shaky ground” before entering the meeting vividly illustrate her feeling of gravity and unsettling urgency about this transitional moment. No man’s land is looming in the horizon, representing the fear of her child being stranded in an uncertain state with no clear path forward, no place to belong. Mia’s questions “There must be some sort of *shelf* if you can’t get into the program? Isn’t there something *else*?” further illustrate the dreaded emptiness of this imagined future. This is the land you go to if you do not fit (Garland-Thomson, 2011) or belong to any of the existing “shelves.” For Mia, it is an imagined future land where there seem to be no open possibilities for Leo to unfold his being or possible becoming, and certainly not to “feel at home.” In other words, No man’s land has the characteristics of a non-dwelling place.

Knowing that Leo might not be able to complete the specialized education program, Mia is even more concerned about the prospect of not getting a plan. Such a scenario carries the risks of another “heavy fall,” as Mia expresses it, referring to an even more dreaded horizon of a ‘non-becoming’ in its most extreme form, the culmination of mental distress, which last time entailed a suicide attempt—the bursting balloon. With a “plan” however, Mia hopes to replace the uncertain horizon of No man’s land with a way forward that is more certain and imaginable. For Mia, this is a project of negotiating a transition from “nowhere to somewhere” (Ginsburg & Rapp, 2024, p. 130). While Mia knows that Leo, as he is now, cannot become a professional firefighter, the program has nonetheless offered both her and Leo a significant discovery—that places do exist where Leo can thrive and unfold his potential. As Mia also puts it at the meeting, “He’s got what it takes—when he’s met the right way.” This echoes the profound sense of “at-stakeness” experienced by parents of autistic children who do not follow typical developmental paths and who face potentially ominous futures (Mattingly, 2017). Similarly, what Mia responds to here is an urgent demand to secure a future dwelling for Leo—a demand with ethical, existential, and ontological stakes.

Mia’s way of addressing the threat of No man’s land at the meeting seems not only a response to a dreaded future scenario but also to Leo’s current situation, where he spends much of his time in his room and does not attend school. As Mia puts it, “He *already* feels he is being excluded, lost.” In this sense, for Mia, No Man’s Land is not only a dreaded future horizon—it is, in some sense, already present. And it even has a materiality that is, Leo’s room—the place where he “won’t get ready” for adulthood, as Mia says, and where he is “*already*” excluded and lost. In this sense, No man’s land takes form as an emic space-time concept.

Conceptualized as a developmental disorder, autism challenges dominant notions of temporality—the linear, progressive temporal reasoning of modern society—a dynamic that also tends to carry with it a margin of spatial exclusion—a “space of belatedness” (Jensen, 2017, p. 86). Leo’s room, embodying both present and future characteristics of No man’s land, captures the temporal and spatial aspects of this dynamic, highlighting how the experience of autism can create a sense of being out of sync with the conventional flow of time and place.

This argument resonates with research linking autism and “vicious cycles” of development (Fein, 2015b). The social navigational challenges that being autistic can entail lead to exclusion from the flow of social life, resulting in amplification of characteristics considered to be deviant, which in turn worsen the cycles of exclusion and atypical development (Fein, 2015b). Leo’s case seems to reflect this dynamic, while the transitional moment he faces also reflects a vicious cycle linked to the notion of No man’s land with its present and future dimensions of constrained possibilities for becoming. One could say that Leo’s non-dwelling experience in his school life, along with his strategies of running home and staying in his room, not only foreshadows but also affects the potential future horizon of a No man’s land. As Mia says before we enter the meeting at the municipality: “It seems like the consequences of poor school choices over time are finally taking effect.” As it emerges ethnographically in this paper, No Man’s Land reflects not only uncertainty about whether Leo can be enrolled in a particular educational track, it also reflects a threatening horizon that a parent battles to hold off—a future of non-becoming and non-dwelling. No man’s land signals a broader existential concern: the urgent demand families face to search for ways to dwell in present and future horizons during the coming-of-age process.

## Conclusion

In this paper, taking the case of Leo and his family as a point of departure, I have offered an ethnographically grounded critical phenomenological exploration of what it means to come of age for autistic adolescents and their families. For Leo, coming-of-age involves a journey that is far from linear, but instead a bumpy journey of past and present challenges with school attendance, experiences of alienation, and the task of reimagining and searching for alternative paths toward adulthood than the typical. Leo’s case illustrates the difficulties many autistic adolescents and their families face in finding a place to belong and a way to become in a world often operating under the quasi-transcendentals of neurotypicality and chrono-normativity. Two contrasting social spaces in Leo’s present life—school and the fire-fighting world—not only reveal the mechanisms shaping his everyday experiences. These spaces are linked to imagined, existential and bureaucratic issues related to coming-of-age. They illustrate how his sense of belonging, selfhood, and possibilities to unfold are contingent on social context. The fire station in particular demonstrates life’s potentiality—the possibility of becoming otherwise. While coming-of-age issues may not typically align with the phenomenological notion of dwelling, I have demonstrated how dwelling has offered a lens for exploring and emphasizing the critical stakes Leo and his family face as part of this transition—in both present and future horizons. They need to find alternative paths where Leo can belong and unfold his being, as the horizon of No man’s land looms threateningly. I suggest that, for some autistic adolescents and their families, coming-of-age poses an intensified dwelling problem—one that spurs the urgent to imagine and find ways of dwelling in both practical but indeed in existential terms, that is, a search for ways to feel at home in the world.

## Footnotes

1 Acknowledging the complex debate around autism terminology, this paper adopts identity-first language to align with my interlocutors’ preferences. When discussing autism more broadly, I also use identity-first language in accordance with the dominant preferences of autism stakeholder groups (Belek, 2019; Taboas et al., 2023).

2 Several anthropologists and philosophers have taken up the notion of dwelling in explorations of relations between humans, their (natural) environment (Ingold, 2000), and animals (Latimer, 2013); of environmental ethics (Guenther, 2002) and how humans dwell in the spectral contours of ‘what-might-have-been’ (Louw, 2025). Michael Jackson attends to dwelling as an existential question of being ‘at home in the world’—a matter of balancing active and passive, autonomous and anonymous modes of being and a sense of existential control and connectedness, where what we do and say seems to matter, and where there is a balanced reciprocity between our self and our environment (1995, 2002).

3 For Zigon, dwelling is inseparable from ethics. Recognizing that ethics and morality are central and contested themes in anthropology (Mattingly & Throop, 2024) including Zigon’s critique of ordinary ethics (Zigon, 2014), in this paper, I can only allude to the ethical dimension of dwelling in the family’s ways of navigating coming-of-age, but without pursuing it in depth.

4 While I find myself hesitant to strictly follow this somewhat idealistic notion of dwelling as the possibility of never being pre-limited within a presumed totality (will there not always be some kind of limiting conditions set by a pre-assumed totality?), I find this notion of dwelling helpful to attend to the existential and ethical—even ontological—at-stakeness autistic adolescents and their families face as they navigate the present and future in a world predominated by neurotypicality—where the possibilities to be and develop outside dominant frameworks of typicality are not always open. It helps us understand the profound impact of environments that limit one’s ability to feel at home in a world and to become ‘otherwise.’

## References

[CR1] Bagatell, N. (2010). From cure to community: Transforming notions of Autism. *Ethos,**38*(1), 33–55. 10.1111/j.1548-1352.2009.01080.x

[CR2] Bagatell, N. (2016). The routines and occupations of families with adolescents with Autism Spectrum Disorders. *Focus on Autism and Other Developmental Disabilities,**31*(1), 49–59. 10.1177/1088357615587503

[CR3] Belek, B. (2019). Autism. In F. Stein, M. Candea, H. Diemberger, S. Lazar, J. Robbins & J. Robbins (Eds.), *Cambridge encyclopedia of anthropology*. University of Cambridge. 10.29164/19aut

[CR4] Britta, Dumstrei (2023) *Skolevægring Forskning i Pædagogers Profession og Uddannelse,**7*(1) 9-10.10.7146/fppu.v7i1.136706

[CR5] Fein, E. (2015a). Making meaningful worlds: Role-playing subcultures and the Autism Spectrum. *Culture, Medicine and Psychiatry,**39*(2), 299–321. 10.1007/s11013-015-9443-x 25812848 10.1007/s11013-015-9443-x PMC4457285

[CR6] Fein, E. (2015b). No one has to be your friend”: Asperger’s Syndrome and the vicious cycle of social disorder in late modern identity markets. *Ethos,**43*(1), 82–107. 10.1111/etho.12073

[CR7] Garland-Thomson, R. (2011). Misfits: A feminist materialist disability concept. *Hypatia,**26*(3), 591–609. 10.1111/j.1527-2001.2011.01206.x

[CR8] Garland-Thomson, R. (2024). What misfitting makes. *Puncta*, *7*(1), pp. 5–22. 10.61372/pjcp.v7i1.2

[CR9] Ginsburg, F. &. Rapp, R., (2024). *Disability worlds*. Duke University Press. 10.1353/book.123468

[CR10] Grinker, R. R. (2020). Autism, “stigma,” disability a shifting historical terrain. *Current Anthropology,**61*(S21), 55–67. 10.1086/705748

[CR11] Guenther, L. (2002). Towards a phenomenology of dwelling. *Canadian Journal of Environmental Education,**7*(2), 38.

[CR12] Guenther, L. (2021). Six senses of critique for critical phenomenology. *Puncta,**4*(2), 5–23. 10.5399/PJCP.v4i2.2

[CR13] Ingold, T. (2000)* The Perception of the Environment: Essays on Livelihood, Dwelling and Skill*. Routledge, New York. 10.4324/9780203466025

[CR14] Jackson, M. (1995). *At home in the world*. Duke University Press.

[CR15] Jackson, M. (2002). *Politics of storytelling : Violence, transgresion and intersubjectivity*. Museum Tusculanum Press.

[CR16] Jensen, V. S. (2017). Performing autism through a layered account. *Departures in Critical Qualitative Research,**6*(1), 72–94. 10.1525/dcqr.2017.6.1.72

[CR17] Latimer, J. (2013). Being alongside: Rethinking relations amongst different kinds. *Theory, Culture & Society,**30*(8), 77–104. 10.1177/0263276413500078

[CR18] Louw, M. (2025). Ghosts of a different present: Specters of possibility in the lives of older Kyrgyz Muslims. *Journal of the Royal Anthropological Institute,**31*(S1), 57–72.

[CR19] Manning, E. (2016). The minor gesture. Duke University Press.

[CR20] Mattingly, C. (2017). Autism and the ethics of care: A phenomenological investigation into the contagion of nothing. *Ethos,**45*(2), 250–270. 10.1111/etho.12164

[CR21] Mattingly, C. & Keeney Parks, S. Chapter 3: Haunted by the Future Autism and the Spectre of Prison – Configuring Race and Disability in the African American Community. *Configuring Contagion: Ethnographies of Biosocial Epidemics*, edited by Lotte Meinert and Jens Seeberg, New York, Oxford: Berghahn Books, 2022, pp. 68-99.10.1515/9781800733053-005

[CR22] Mattingly, C., & Throop, J. (2024). The anthropology of ethics and morality. *Annual Review of Anthropology,**47*, 475–492. 10.1146/annurev-anthro-102317

[CR23] Ochs, E., & Solomon, O. (2010). Autistic sociality. *Ethos,**38*(1), 69–92. 10.1111/j.1548-1352.2009.01082.x

[CR24] Prince, D. E. (2010). An exceptional path: An ethnographic narrative reflecting on autistic parenthood from evolutionary, cultural, and spiritual perspectives. *Ethos,**38*(1), 56–68. 10.1111/j.1548-1352.2009.01081.x

[CR25] PwC & VIVE. (2020). *Unge med autisme og over- gangen til et selvstændigt liv Litteraturkortlægning - notat*.

[CR26] Rapp, R., & Ginsburg, F. (2011). Reverberations: Disability and the new kinship imaginary. *Anthropological Quarterly,**84*(2), 380–410. 10.1353/anq.2011.0030

[CR27] Reynolds, J., & Weiss, G. (2024). Fits and misfits. *Puncta,**7*(1), 1–4.

[CR28] Rodogno, R., & Krause-Jensen, K. (2023). Shame on Wrong Planet. In A. Fussi & Raffaele Rodogno (Eds.), *The Moral Psychology of Shame*. (pp.221-243). Rowman & Littlefield Publishers. https://www.researchgate.net/publication/370445236

[CR29] Sacks, O. (1996). *An anthropologist on mars: Seven paradoxical tales*. Vintage. 10.4103/0028-3886.258001 31085904

[CR30] Skovbo Rasmussen, P., & Pagsberg, A. K. (2019). Customizing methodological approaches in qualitative research on vulnerable children with autism spectrum disorders. *Societies,**9*(4), 1–16. 10.3390/soc9040075

[CR31] Taboas, A., Doepke, K., & Zimmerman, C. (2023). Preferences for identity-first versus person-first language in a US sample of autism stakeholders. *Autism,**27*(2), 565–570. 10.1177/13623613221130845 36237135 10.1177/13623613221130845

[CR32] Zigon, J. (2014). An ethics of dwelling and a politics of world-building: A critical response to ordinary ethics. *Journal of the Royal Anthropological Institute,**20*(4), 746–764. 10.1111/1467-9655.12133

